# Comparison of Treated Mean Intraocular Pressure in Stable Glaucoma with Different Severity in Vietnam

**DOI:** 10.5005/jp-journals-10008-1153

**Published:** 2014-01-16

**Authors:** Nguyen Thi Ha Thanh

**Affiliations:** Master of Ophthalmology, Department of Glaucoma, National Institute of Ophthalmology Vietnam

**Keywords:** Intraocular pressure, Stable glaucoma, Stage, Medication.

## Abstract

**Purpose:** To compare stable glaucoma with different severity in a Vietnamese population in regard to mean intraocular pressure (IOP) and number of medications used.

**Materials and methods:** A total of 116 eyes from 68 patients with medically treated glaucoma were prospectively enrolled at a single center and subjected to automated perimetry every 3 months for at least 9 months. Glaucoma progression was identifed according to early manifest glaucoma trial criterion using glaucoma progression analysis software. Eyes in which no progression was identifed were staged for glaucoma severity using field criteria (mild MD ≥ 6 dB, moderate MD –6 to –12 dB, advanced MD ≥ 12 dB, end-stage central island only). Groups were compared in terms of mean IOP and number of medications used. Statistical analysis was performed using SPSS v16.0.

**Results:** A total of 109 eyes displayed no evidence of pro gres-sion during the study period. Pretreatment mean IOP for mild, moderate, severe and end-stage glaucoma was 28.2 ± 1.4, 28.8 ± 1.6, 29.1 ± 1.8, and 28.6 ± 0.8 mm Hg. The mean IOP of all 109 eyes during follow-up was 16.8 ± 1.4 mm Hg (95% conf dence interval = 15.4 ± 18.2 mm Hg). Mild, moderate, advan ced, and end-stage glaucoma had mean IOP of 17.5 ± 1.2, 16.9 ± 1.3, 15.8 ± 0.9 and 15.5 ± 1.1 mm Hg. The mean IOP of mild stage was significantly higher than advanced and end-stage (t-test, p < 0.001). Also, the mean IOP of moderate glaucoma was significantly higher than advanced and end-stage glaucoma (t-test, p < 0.05). Number of medications had no signi ficant difference among these glaucoma stages (chi-square test, p > 0.05).

**Conclusion:** Reached IOP lowering contributes to glaucoma stabilization especially in late stages. To maintain stable glaucoma, there was no difference in medical procedure of glaucoma stages.

**How to cite this article:** Thanh NTH. Comparison of Treated Mean Intraocular Pressure in Stable Glaucoma with Different Severity in Vietnam. J Current Glau Prac 2014;8(1):7-9.

## INTRODUCTION

Elevated intraocular pressure (IOP) is an important risk factor for the development or progression of glaucomatous optic neuropathy. As such, IOP reduction is an important strategy to slow or halt glaucoma progression and irreversible visual impairment.^1,2^

First line medical treatment for lowering IOP is mono therapy with either a topical prostaglandin analog or a β-adrenergic antagonist (β-blocker). However, many patients eventually require adjunctive therapy to achieve their target IOP and maintain stable glaucoma.^3^

Although, many studies have reported on IOP in stable glaucoma and how this was achieved, no studies have examined this in a Vietnamese population. Therefore, the aim of this study was to compare stable glaucoma with different severity in regard to mean IOP and number of medications used.

## MATERIALS AND METHODS

This prospective study was conducted within the glaucoma department at the Vietnam National Institute of Ophthalmology (VNIO) from August, 2011 to August, 2013 and approved by the VNIO research ethics committee.

### Participants

Participants were included in the study if they were aged between 18 and 70 years and had established primary open angle glaucoma (POAG) on one or more topical medical therapies. Exclusion criteria included secondary open angle or angle closure glaucoma, a history of previous laser trabeculoplasty or glaucoma fltration surgery, if they were not able to give informed consent, if they could not perform automated perimetry reliably (>3 fixation losses, >20% false positive, and >20% false negative), or if coexisting ocular conditions including previous trauma, significant cataract, corneal disease, or retinopathy. Participants on systemic medications that may infuence IOP(e.g. oral β-blocker) were also excluded.

Primary open angle glaucoma was defined by the presence of characteristic optic nerve damage with a corres-ponding visual field defect in the presence of an open normal appearing iridocorneal angle and the absence of known secondary causes of elevated IOP.

### Follow-up Protocol

First-line IOP lowering treatment consisted of either topical prostaglandin analog or β-blocker monotherapy. If IOP was increased above target, adjunctive therapy was added in a stepwise sequential manner until target IOP was reached.

Participants were assessed for visual acuity, IOP using Goldmann applanation tonometry, slit lamp biomicroscopy, gonioscopy and pachymetry. The retinal nerve fiber layer thickness was measured using by Cirrus optical coherence tomography.

Visual Fields

Automated perimetry was performed at baseline and at 3 monthly follow up intervals for a minimum 9 months using the Humphrey perimeter. Baseline perimetry consisted of two tests performed 1 week apart. Perimetry was performed in a dark room under the supervision of visual field specialist. Reliability indices were monitored and were considered high if >3 fixation losses, >20% false positives, or >20% false negatives were detected. In this situation, the testing was cancelled, participants reinstructed then testing commenced again.

Glaucoma progression was identifed according to early manifest glaucoma trial (EMGT) criterion using glaucoma progression analysis software. The patient had completed at least five visual field tests, including the two baseline visual field and three tests follow up. Mean deviation (MD), pattern standard deviation (PSD), visual field index (VFI), and glaucoma progression analysis (GPA) progression symbols were recorded. Possible progression required at least three test points deteriorated p < 0.05 repeated in two visual field tests. Likely progression required at least three test points deterio rated p < 0.05 repeated in three visual field tests.^4-6^ Eyes in which no progression was identifed were staged for glaucoma severity using field criteria (mild MD ≥ 6dB, moderate MD –6 to –12 dB, advanced MD ≥ 12 dB, end stage central island only).

**Graph 1:**
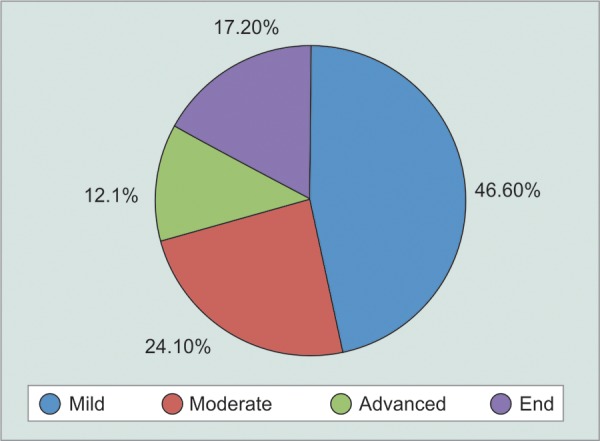
Different severity

**Graph 2:**
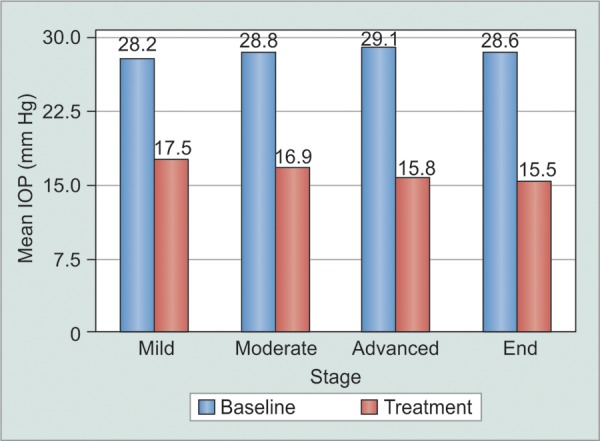
Mean intraocular pressure of different severity

**Table 1:** **Table 1:** Age of patients

*Age (years)*		*No. of patients (%)*	
<40		24 (35.3)	
40-60		38 (55.9)	
>60		6 (8.8)	

**Table 2:** **Table 2:** Type of medications

*Type of medications*		*No. of eyes (%)*	
Prostaglandin		50 (43.1)	
β-blocker		18 (15.5)	
Prostaglandin + β-blocker		22 (19)	
Brimonidine + β-blocker		3 (2.6)	
Prostaglandin + β-blocker + Azopt		17 (14.7)	
Prostaglandin + β-blocker + Azopt + Alphagan		6 (5.1)	
Total		116 (100)	

**Table 3:** **Table 3:** Number of medications

*No. of medications*		*No. of eyes (%)*	
1		68 (58.6)	
2		25 (21.5)	
3		17 (14.7)	
4		6 (5.2)	
Total		116 (100)	

Statistical Analysis

The statistical analysis systems software (SPSS v16.0) was used for all statistical analyses. Differences between others stage were analyzed using Student's t-test and chi-square test. Differences were statistically significant at p < 0.05.

**Table 4:** **Table 4:** Number of medications in different severity

*Stages*		*1 medication(%)*		*2 medications(%)*		*3 medications(%)*		*4 medications(%)*		*Total(%)*	
Mild		35 (67.3)		9 (17.3)		6 (11.6)		2 (3.8)		52 (100)	
Moderate		17 (63)		7 (25.9)		3 (11.1)		0		27 (100)	
Advanced		9 (75)		2 (16.7)		0		1 (8.3)		12 (100)	
End		5 (27.8)		6 (33.3)		6 (33.3)		1 (5.6)		18 (100)	

Number of medications had no significant difference among these stable glaucoma stages (Chi-square test, p > 0.05)

## RESULTS

A total of 116 eyes of 68 POAG patients were initially enrolled. Of these, 109 eyes displayed, no evidence of glaucoma progression using EMGT criteria and were included in the analysis. The mean and range of age was 46.2 ± 22.3 years with most patients aged 40 years or older (Table 1).

The mean and range of follow-up was 16.3 ± 5.7 months. The mean number of visual field tests performed was 7.2 ± 2.1. The majority of eyes had mild POAG (Graph 1).

Prostaglandin monotherapy was the most common treatment (43.1%) (Table 2). Most of the eyes had one medication (58.6%) (Table 3). Although the ratios of two and three medications were high in the end stage, the number of medications had no significant difference among these stable glaucoma stages (Table 4).

The mean pretreatment IOP of all eyes was 28.4 ± 1.3 mm Hg. Mean IOP at final follow up of all eyes on treatment was 16.8 ± 1.4 mm Hg. The mean IOP of mild stage was significantly higher than advanced and end stage (t-test, p < 0.001). Also, the mean IOP of moderate glaucoma was significantly higher than advanced and end stage glaucoma (t-test, p < 0.05) (Graph 2).

## DISCUSSION

This study assessed the mean treated IOP of 109 eyes with no glaucoma progression using EMGT visual field criteria in a Vietnamese population. The study found that mean IOP was significantly lower in stable advanced or end stage glaucoma compared to stable moderate glaucoma or stable mild glaucoma.

The findings of this study are consistent with the recom mendation of the world glaucoma association (WGA) suggesting that target IOP should be progressively lower for increasing disease severity (safe IOP of mild stage is ≤ 21 mm Hg, of moderate is ≤18 mm Hg, of advanced is ≤15 mm Hg, of end is ≤12 mm Hg),^7^ although it is interesting to note that the mean IOP reported in this study does not exactly match the recommended levels by the WGA.

To achieve target IOP, the first choice monotherapy is usually a prostaglandin analog or β-blocker depending on availability, patient suitability, and cost. In this study, almost 60% of all eyes with stable glaucoma achieved this with mono therapy and up to 75% of mild to severe glaucoma remained stable on a single agent. This fell dramatically to 27.8% for end stage glaucoma which likely refects a desire to achieve a much lower IOP in this stage of disease.

When target IOP is not achieved, switching or adding an agent is the logical next step. In eyes that respond insufficiently to initial β-blocker or a prostaglandin analog, a fixed combination therapy that consists brimonidine/timolol, or prostaglandin analog/timolol can be considered because of ease of use and cost advantages. In our population, this treatment strategy was seen in 21.6% of eyes.

## CONCLUSION

It is essential to detect and monitor glaucoma progression to ensure that the treated IOP is safe for patients. IOP reducing is important to maintain stable glaucoma, with lower IOP required as the glaucoma becomes more advanced.
